# Reduced Models of Point Vortex Systems

**DOI:** 10.3390/e20120914

**Published:** 2018-11-30

**Authors:** Jonathan Maack, Bruce Turkington

**Affiliations:** Department of Mathematics and Statistics, University of Massachusetts Amherst, Amherst, MA 01003, USA

**Keywords:** vortex dynamics, nonequilibrium statistical mechanics, quasi-geostrophic equations, coherent structures

## Abstract

Nonequilibrium statistical models of point vortex systems are constructed using an optimal closure method, and these models are employed to approximate the relaxation toward equilibrium of systems governed by the two-dimensional Euler equations, as well as the quasi-geostrophic equations for either single-layer or two-layer flows. Optimal closure refers to a general method of reduction for Hamiltonian systems, in which macroscopic states are required to belong to a parametric family of distributions on phase space. In the case of point vortex ensembles, the macroscopic variables describe the spatially coarse-grained vorticity. Dynamical closure in terms of those macrostates is obtained by optimizing over paths in the parameter space of the reduced model subject to the constraints imposed by conserved quantities. This optimization minimizes a cost functional that quantifies the rate of information loss due to model reduction, meaning that an optimal path represents a macroscopic evolution that is most compatible with the microscopic dynamics in an information-theoretic sense. A near-equilibrium linearization of this method is used to derive dissipative equations for the low-order spatial moments of ensembles of point vortices in the plane. These severely reduced models describe the late-stage evolution of isolated coherent structures in two-dimensional and geostrophic turbulence. For single-layer dynamics, they approximate the relaxation of initially distorted structures toward axisymmetric equilibrium states. For two-layer dynamics, they predict the rate of energy transfer in baroclinically perturbed structures returning to stable barotropic states. Comparisons against direct numerical simulations of the fully-resolved many-vortex dynamics validate the predictive capacity of these reduced models.

## 1. Introduction

The equilibrium statistical mechanics of point vortex systems now exists as a mature theory. Onsager provided the first insight that equilibrium theory is able to explain the organization of many-vortex systems into coherent structures [1]. He also indicated that these systems have novel thermodynamic properties, most notably that they allow equilibria with negative absolute temperature and that the usual equivalence of ensembles between microcanonical and canonical distributions may break down. In the decades since his prescient work, not only have these properties been established theoretically and verified numerically, but it has been understood that, due to the long-range nature of the interactions between point vortices, the appropriately-scaled continuum limit is an exact mean-field theory [2,3,4,5]. Consequently, the thermodynamic behavior of equilibrium states is completely determined by a mean-field equation, whose solutions are steady two-dimensional flows having a special functional relation between mean vorticity and streamfunction, which is derived from the populations of the vortex strengths (circulations) in the statistical ensemble [6,7,8].

Numerical and experimental studies of freely-decaying two-dimensional turbulence have repeatedly demonstrated that coherent structures emerge and persist within the vorticity fluctuations and filaments of high Reynolds number flow and that these vortical structures eventually dominate the large scales of the flow [9,10,11,12]. These coherent structures have naturally been proposed as realizations of the equilibrium statistical states of an underlying point vortex dynamics; for instance, direct numerical simulations in doubly-periodic geometry tend to produce the dipolar end states predicted by the mean-field theory [13]. More recently, alternative statistical equilibrium theories have been developed from other discretizations of the continuum equations for an incompressible, inviscid fluid, which respect invariants that the point vortex idealization violates [14,15,16]. From the point of view of fluid mechanical outcomes, however, these more intricate theories are similar to the point vortex theory, in that they also derive special mean-field equations satisfied by their equilibrium states. These theories, when extended to include quasi-geostrophic dynamics, have been shown to realize interesting coherent structures such as the zonal jets and embedded Great Red Spot on Jupiter [7,17,18].

By contrast, there is no comparable theory of the nonequilibrium behavior of vortex ensembles, which could furnish models of the unsteady behavior of coarse-grained states in two-dimensional or quasi-geostrophic turbulence. This is not surprising in view of the large conceptual gap between equilibrium and nonequilibrium statistical mechanics themselves. Whereas statistical equilibrium is conceived in the unified framework of Gibbsian ensembles built from exactly conserved quantities, nonequilibrium descriptions depend upon some selection of relevant, or resolved, variables, which are not conserved but are intended to produce a coarse-grained dynamics. Typically the feasibility of such a selection depends on some separation of scales of motion, which may or may not be satisfied in problems of interest. One systematic approach is through kinetic theory and its hierarchy of reduced distribution functions. In this context modeling becomes a choice of a truncation of the hierarchy and a concomitant closure hypothesis, carrying with it restrictions on its range of validity [19]. An interesting approach to vortex systems through kinetic theory is developed in [20].

In this paper, we address the nonequilibrium statistical mechanics of point vortex systems by means of a less traditional method of model reduction, which we call “optimal closure” [21,22]. Some previous publications have applied this method to other prototypical problems in statistical fluid dynamics [23,24,25,26]. Our goal is to apply the optimal closure method to model the relaxation of near-equilibrium vortical structures toward equilibrium. Specifically, we seek to extend the range of the mean-field theory known for equilibrium states to a comparable nonequilibrium mean-field theory of near-equilibrium relaxation.

The principal merit of the optimal closure method is that, given a reduction defined by some selected macroscopic observables, it extracts the intrinsic dissipative equations for the macrostates directly from the underlying microscopic dynamics. The accuracy of the reduced dynamics depends, of course, upon the choice of the macroscopic description. Since a practical model reduction relegates many degrees of freedom to a statistical description, there is no guarantee that the derived macroscopic evolution will agree quantitatively with the ensemble-averaged behavior of the full-resolved microscopic dynamics. The problem addressed in the present paper, therefore, offers a useful test of the optimal closure method, since it concerns a severe coarse-graining of point vortex dynamics. For this reason the paper first develops the reduced model theoretically, and then proceeds to validate it against direct numerical simulations of ensembles.

For simplicity, we restrict our analysis to isolated structures, that is, ensembles of many, like-signed, equal circulation, point vortices in the entire plane, whose equilibrium states are single macrovortices centered at the origin. The severe coarse-graining we impose takes the macroscopic variables to be the first- or second-order spatial moments of the vorticity. These moments, together with the conserved energy and circulation, describe the overall “shape” of the vortex ensemble. For instance, an initially elliptical structure develops spiral arms that wrap around its center and eventually homogenize into an axisymmetric end state. Our reduced model of this equilibration uses only the second-order spatial moments of the vorticity around the center to follow the evolving structure; heuristically, the reduced model replaces the intricate configuration of point vortices by an effective elliptical distribution of vorticity.

In the first half of the paper we develop the optimal closure theory of point vortex systems governed by inviscid, incompressible Euler equations in the plane. In the second half we generalize that theory to quasi-geostrophic dynamics, first for barotropic single-layer flows and then for baroclinic two-layer flows. For these simplified geophysical fluids, we conduct numerical experiments to test the accuracy of the reduced models. We especially draw attention to the predicted rates of relaxation of vortical structures and the dependence of those rates on the key parameters in the quasi-geostrophic equations. Indeed, the useful outcome of such severe model reduction and closure is to approximate gross features and functional dependences with much greater computational efficiency than is required by direct numerical studies of the full dynamics.

## 2. Statistical Mechanics of Point Vortices

The streamfunction-vorticity formulation of the incompressible, inviscid, two-dimensional Euler equation in the plane is

(1a) $$\begin{array}{c} \partial_{t}\zeta +\left[\zeta ,\psi \right]=0, \end{array}$$

(1b) $$\begin{array}{c} -\Delta \psi =\zeta , \end{array}$$

where

$$\left[A,B\right]=\frac{\partial A}{\partial x}\frac{\partial B}{\partial y}-\frac{\partial A}{\partial y}\frac{\partial B}{\partial x}.$$

denotes the Poisson bracket on $R^{2}$. (Throughout we consider an appropriately nondimensionalized flow problem, in which the physical variables *x* and *y* are scaled by *L*, ζ by $T^{-1}$ and the fluid density by $ML^{-2}$, using characteristic length, time and mass scales, L,T,M; thus, the fluid density is unity in our formulation.) The scalar conservation law (1a), which states that the vorticity ζ(x,t) is constant along all flow trajectories, $\dot{x}=\partial \psi /\partial y,\;\;\dot{y}=-\partial \psi /\partial x$, is the distinguishing property of two-dimensional flow [27,28]. In particular, it implies the conservation of $\Gamma \left(\zeta \right)=\int_{R^{2}}\zeta \;dx$, which by Stokes’ formula coincides with the total circulation, as well as the conservation of all higher moments of the vorticity (general enstrophy integrals). The continuum dynamics (Section 2) has a (generalized) Hamiltonian structure, and its Hamiltonian is a quadratic functional of ζ [8,28]; namely,

(2) $$H\left(\zeta \right)=\frac{1}{2}\int_{R^{2}}\psi \;\zeta \;dx\;.$$

The translational and rotational symmetries of the domain imply two additional invariants; namely, the first and (radial) second spatial moments of vorticity,

(3) $$B\left(\zeta \right)=\int_{R^{2}}x\;\zeta \;dx,\;\;\;\;\;\;\;\;I\left(\zeta \right)=\int_{R^{2}}{\left|x\right|}^{2}\zeta \;dx.$$

We shall refer to the invariants *H*, *B* and *I* as the energy, linear impulse and angular impulse [27]. Technically these three invariants are the finite parts of the total kinetic energy, linear momentum and angular momentum, respectively, which are divergent for a flow in the plane induced by a compactly supported vorticity ζ, since its velocity field decays like ${\Gamma |x|}^{-1}$ as |x|→∞.

A point vortex dynamics occurs when the vorticity distribution is sharply concentrated at *N* points:

(4) $$\zeta^{N}\left(x,t\right)=\sum_{i=1}^{N}\gamma_{i}\delta \left(x-x_{i}\left(t\right)\right)$$

where $\gamma_{i}$ is the vortex strength (or circulation) at the point $x_{i}$, the location of the $i^{th}$ vortex; δ(x) is the unit delta function on $R^{2}$. The Euler equations then collapse to advection of the point vortices by the induced flow, whose streamfunction is

(5) $$\psi^{N}\left(x,t\right)=-\frac{1}{2\pi }\sum_{i=1}^{N}\gamma_{i}\log\left|x-x_{i}\left(t\right)\right|\;.$$

This classical reduction of the continuum equations to a finite-dimensional dynamical system for the point vortex locations ignores the self-induced velocity field of each vortex [29]. While intuitively evident, this desingularization can be justified analytically in the point vortex limit of the continuum dynamics under some restrictions [28]. The point vortex dynamics may be expressed as a canonical Hamiltonian system in the variables, $q_{i}=\sqrt{\gamma_{i}}x_{i},\;p_{i}=\sqrt{\gamma_{i}}y_{i}$, after rewriting the desingularized energy,

(6) $$H^{N}\left(x_{1},\ldots ,x_{N}\right)=-\frac{1}{4\pi }\sum_{i,j=1,i\neq j}^{N}\gamma_{i}\gamma_{j}\log\left|x_{i}-x_{j}\right|\;,$$

as a function $H^{N}\left(q_{1},p_{1},\ldots ,q_{n},p_{n}\right)$ [28,29].

Our interest centers exclusively on systems of identical vortices. Accordingly, we set $\gamma_{i}=1/N$, so that $\Gamma (\zeta^{N})=1$. To express the point vortex dynamics in a Hamiltonian form with respect to the spatial coordinates $x_{i}=\left(x_{i},y_{i}\right)$ themselves, we employ the rescaled Poisson bracket,

(7) $$\left\{F,G\right\}\;=\;N\sum_{i=1}^{N}{\left[\;F,G\;\right]}_{\left(x_{i},y_{i}\right)}\;;$$

*F* and *G* denote generic functions on the phase space $R^{2N}$ of the system, and the planar bracket acts on their $x_{i}$ variables separately. The point vortex dynamics is then equivalent to the family of identities:

$$\frac{dF}{dt}\;=\;\left\{F,H^{N}\right\}\;\;\;\;\;\mathrm{for}\;\mathrm{all}\;\left(\mathrm{smooth}\right)\;\text{real-valued}\;\mathrm{functions}\;F=F\left(x_{1},\ldots x_{n}\right)\;.$$

Besides $H^{N}$ and $\Gamma^{N}=1$, this Hamiltonian dynamics conserves the linear and angular impulses, respectively,

$$B^{N}=\frac{1}{N}\sum_{i=1}^{N}x_{i}\;=\;0\;,\;\;\;\;\;\;I^{N}=\frac{1}{N}\sum_{i=1}^{N}{\left|x_{i}\right|}^{2}\;,$$

after centering the vortex system at the origin.

Statistical equilibrium theory invokes the ergodic hypothesis and considers invariant probability distributions on the phase space constrained by the known invariants [30,31]. In particular, the canonical Gibbs density,

(8) $$\rho^{N}\left(x_{1},\ldots ,x_{N}\right)\;=\;\exp\left(-N\beta H^{N}-N\alpha I^{N}-\phi \left(\beta ,\alpha ,N\right)\right)\;,$$

is parameterized by constants β and α conjugate to the ensemble mean values of energy and angular impulse, respectively, $\langle H^{N}\rangle =E$ and $\langle I^{N}\rangle =L^{2}$; ϕ(β,α,N) normalizes the probability density $\rho^{N}$. Here and throughout our subsequent discussion, the angle brackets 〈·〉 denote average, or expectation, with respect to a specified density; in this case $\rho^{N}$. The invariant $B^{N}$ is able to be ignored, as the centering constraint $\langle B^{N}\rangle =0$ is automatically satisfied.

In (8) the canonical parameters are scaled by *N* in anticipation of the appropriate continuum limit, in which *E* and $L^{2}$ are fixed and finite as N→∞. In that scaled limit the theory of large deviations characterizes the behavior of the empirical measure $\zeta^{N}$ in (4), that is, the spatial density of point vortices [4]. Specifically, $\zeta^{N}$ tends in the weak sense of measures on $R^{2}$ to the maximizer ζ of the entropic functional

$$S_{\beta ,\alpha }\left[\zeta \right]=-\int_{R^{2}}\zeta \left(x\right)\log\zeta \left(x\right)dx\;-\;\beta H\left(\zeta \right)-\alpha I\left(\zeta \right)$$

over probability densities ζ(x) on $R^{2}$. The same large deviation principle quantifies the likelihood of fluctuations away from the most probable density ζ(x) for large *N*: the likelihood of another state $\tilde{\zeta }$ is exponentially small in *N* with a rate functional equal to $S_{\beta ,\alpha }\left[\zeta \right]-S_{\beta ,\alpha }\left[\tilde{\zeta }\right]\;$. The relevant large deviation theory is explained in [32] and its application to point vortex ensembles is summarized in [8].

The optimality conditions for the equilibrium state, ζ=−Δψ, imply that its streamfunction ψ satisfies the mean-field equation

(9) $$-\Delta \psi =e^{-\beta \psi -{\alpha |x|}^{2}-\mu }\;,$$

for a constant μ adjusted so that ζ is a probability density on $R^{2}$. This mean-field equation is the equilibrium condition for the constrained minimizer ζ of $S_{\beta ,\alpha }$ subject to ∫ζdx=1. Namely, the Lagrange multiplier rule yields

$$\begin{array}{ccc} 0 & = & \delta S_{\beta ,\alpha }-\left(\mu -1\right)\;\delta \int \zeta \;dx\\ & = & -\int \left[\;\log\zeta +1+\beta \psi +{\alpha |x|}^{2}+\mu -1\;\right]\delta \zeta \;dx\;, \end{array}$$

using the multiplier μ−1; since the variation δζ is arbitrary, (9) follows. Thus the most probable state in the continuum limit with finite energy and angular impulse is a special steady solution of the Euler equations, having an exponential dependence of vorticity on streamfunction. That special dependence in the macrostate is a direct consequence of the modeling assumption that the microstate consists of many identical point vortices [28,29].

It is often advantageous to associate these equilibrium flows with the energy and angular impulse values that they attain, and to parametrize branches of solutions to the mean-field Equation (9) by *E* and $L^{2}$ rather than β and α. Of course, this change of perspective is realized by replacing the canonical ensemble by the microcanonical ensemble. Microcanonical equilibrium states are constrained maximizers of entropy:

(10) $$\begin{array}{c} \mathrm{maximize}\;-\int_{R^{2}}\zeta \left(x\right)\log\zeta \left(x\right)dx\;\;\;\;\;\;\;\;\;\mathrm{subject}\;\;\mathrm{to}\\ \frac{1}{2}\int_{R^{2}}\psi \left(x\right)\zeta \left(x\right)dx=E,\;\;\;\;\int_{R^{2}}{\left|x\right|}^{2}\zeta \left(x\right)dx=L^{2},\;\;\;\;\int_{R^{2}}\zeta \left(x\right)dx=1\;; \end{array}$$

β,α,μ−1 are then the Lagrange multipliers associated with the equality constraints, and the maximizer satisfies the mean-field Equation (9). A large deviation analysis similar to that for canonical equilibria is applicable to microcanonical equilibria [8]. An iterative algorithm exists to solve the constrained optimization problem (10) and thus to compute branches of equilibrium states [33]. Using this algorithm it is possible to establish that the maximum entropy is a concave function of the constraint values $E,L^{2}$, and hence that the canonical and microcanonical formulations are equivalent in this particular problem; for vortex systems under other conditions the equivalence of ensembles may break down when β<0 [34,35].

**Figure 1:**
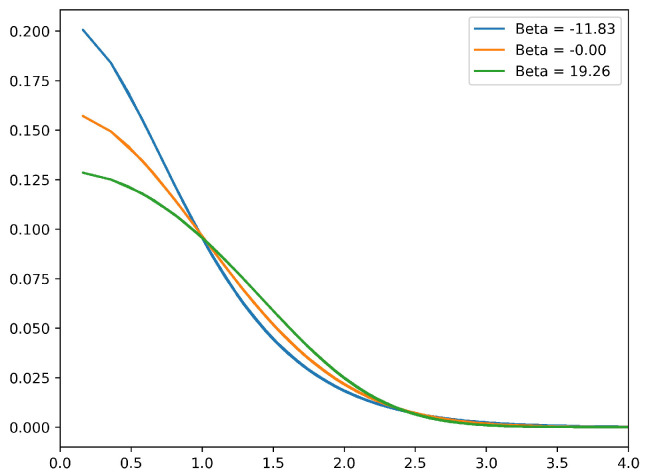
Radial profiles of vorticity for representative equilibrium states with β<0,β=0,β>0.

These equilibrium states are axisymmetric, stable, macrovortices with radially decreasing vorticity. For β=0 they are Gaussian densities, while for β>0 they are less concentrated, and for β<0 they are more concentrated at the origin than a Gaussian. Figure 1 displays three representative radial profiles of the vorticity ζ(r).

## 3. Optimal Closure Method

When we endeavor to construct a nonequilibrium theory of macroscopic states, and closed evolution equations for those states, we necessarily include observables in the macroscopic description that are not exact invariants. Accordingly, each choice of macroscopic observables leads to a corresponding nonequilibrium reduced model. This arbitrariness can be circumvented only when the system under study has a strong separation of time scales, so that its slow variables can form the macroscopic description, and its fast variables can be relegated to a statistical treatment. Even in such a case a unique reduction is only achieved in the limit as the separation of time scales becomes infinite. The current paper therefore adopts a different perspective. We accept that some choice of resolved, macroscopic observables must be made, and that this choice is not unique. Given a chosen set of observables—presumably a reasonable choice from the standpoint of a desired reduced model—the mathematical problem then becomes how to devise a suitable closure in terms of those observables.

We approach this problem using a combination of statistical modeling and information theory. Namely, we construct a parametric family of “trial” probability densities on the phase space of the microscopic dynamics (assumed to be deterministic and Hamiltonian), having parameters that are in a one-to-one correspondence with the mean values of the chosen observables. Within the reduced statistical model defined by that parametric family we attempt to follow the propagation of probability induced by the underlying microscopic dynamics. To do so we quantify the deviation between the exactly propagated density and any feasible evolution of the model density, using a metric derived from the relative entropy, or Kullback-Leibler divergence, between the exact and model densities. Dynamical closure within the reduced model is then determined by minimizing this metric over feasible paths in the parameter space of the model. In this way, we obtain a statistically consistent closure, in which macrostates evolve so as to minimize the rate of information loss due to model reduction.

This optimal closure method of model reduction was introduced by one of the authors in [22]. In that paper a weighted metric is considered, the weights being adjustable parameters in the closure. Subsequent studies have shown, however, that these adjustable weights are unnecessary, and that an intrinsic optimal closure is achieved by setting all weights equal to unity [21,25,26].

In the remainder of this section we summarize the optimal closure method in the general context of reducing a Hamiltonian dynamics onto a finite set of linearly independent observables, $A_{1},\ldots ,A_{m}$. Let *z* denote a generic point in the phase space $R^{2N}$; for instance, in the point vortex system, $z=(x_{1},\ldots ,x_{N})$. For “trial” probability densities on the phase space we take quasi-canonical densities built from the chosen observables, namely,

(11) $$\tilde{\rho }\left(z;\xi \right)\;=\;\exp\left(\;\sum_{k=1}^{m}\xi_{k}A_{k}\left(z\right)-\beta H\left(z\right)-\phi \left(\xi ,\beta \right)\;\right)\;,$$

where $\xi =(\xi_{1},\ldots ,\xi_{m})$ is the parameter vector conjugate to the resolved vector $A=(A_{1},\ldots ,A_{m})$; ϕ(ξ,β) normalizes the probability. The probability densities (11) constitute an exponential family with natural parameters $\xi \in R^{m}$ and −β∈R [36,37]. We are interested in modeling the relaxation of macrostates toward statistical equilibrium, and so we slave β to ξ by imposing the conservation of energy, namely, that 〈H〉=E, where *E* is the equilibrium energy. Here the expectation is taken with respect to the trial density $\tilde{\rho }$. We defer until the next section any specification of the vector observable *A*, except to say that A(z) represents some coarse-graining of the microstate *z*.

The exact propagation of probability on the phase space $R^{2N}$ is expressible formally as

$$\rho \left(t\right)=e^{-(t-t_{0})L}\rho \left(t_{0}\right),\;\;\;\;\;\;\;\;\mathrm{where}\;\;L=\left\{\cdot ,H\right\}\;.$$

Indeed, this statement is equivalent to the Liouville equation,

$$\partial_{t}\rho \;+\;\left\{\rho ,H\right\}\;=\;0\;,$$

which is the foundation of statistical mechanics [19,31,38]. Our closure procedure is based on submitting trial densities $\tilde{\rho }\left(\cdot ;\xi \left(t\right)\right)$ to the Liouville equation and minimizing the mean-square of the resulting residual over parameter paths ξ(t). Equivalently, our closure principle can be viewed in information-theoretic terms by analyzing the Kullback-Leibler divergence between an exactly propagated density and a feasible model density [39,40]. Namely, let $\tilde{\rho }\left(t\right)=\rho \left(t\right)$ at time *t*, and for a short interval of time Δt consider the incremental relative entropy,

$$\begin{array}{ccc} D_{KL}\left(\;e^{-\Delta tL}\rho \left(t\right)\;∥\;\tilde{\rho }\left(t+\Delta t\right)\;\right) & = & \int_{R^{2N}}e^{-\Delta tL}\rho \left(t\right)\;\log\frac{e^{-\Delta tL}\rho \left(t\right)}{\tilde{\rho }\left(t+\Delta t\right)}dz\\ & = & \frac{{\left(\Delta t\right)}^{2}}{2}\langle {\left[\;\left(\partial_{t}+L\right)\log\tilde{\rho }\left(\cdot ;\xi \left(t\right)\right)\right]}^{2}\rangle \;+\;o\left(\;{\left(\Delta t\right)}^{2}\;\right)\;, \end{array}$$

expanding in a Taylor series in Δt; the expectation in the second equality is taken with respect to $\tilde{\rho }\left(\cdot ;\xi \left(t\right)\right)$. The leading order term in this expansion of the relative entropy is an intrinsic metric on the discrepancy between the exact and model densities. In view of this calculation, we define the Liouville residual to be

(12) $$R\;=\;R\left(\xi ,\dot{\xi }\right)=\;\left(\partial_{t}+L\right)\log\tilde{\rho }\left(\cdot ;\xi \left(t\right)\right)\;,$$

on any feasible path ξ(t), and we quantify the local lack-of-fit of the reduced model by

(13) $$𝓛(\xi ,\dot{\xi })=\frac{1}{2}\langle R^{2}\rangle \;.$$

Our optimal closure minimizes the time integral of $𝓛(\xi ,\dot{\xi })$ over feasible parameter paths. These extremal paths realize the slowest rate of information loss, or entropy production, within the reduced model, and thus represent the best fit of the trial densities to the Liouville equation.

The optimality conditions for the closure are conveniently derived by applying Hamilton-Jacobi theory [41,42,43]. That is, we view 𝓛 as a Lagrangian function and introduce its value function (analogous to an action integral),

(14) $$v\left(\bar{\xi },\bar{t}\right)\;=\;\min_{\xi \left(t\right),\xi \left(0\right)=\bar{\xi }}\int_{0}^{\bar{t}}𝓛(\xi ,\dot{\xi })\;dt\;.$$

To the lack-of-fit Lagrangian we associate a lack-of-fit Hamiltonian (not to be confused with that for the microscopic dynamics) and conjugate variables, namely,

(15) $$𝓗(\xi ,\pi )=\max_{\dot{\xi }}\;\sum_{k=1}^{m}\pi_{k}{\dot{\xi }}_{k}\;-\;𝓛(\xi ,\dot{\xi })\;,\;\;\;\;\;\;\;\;\;\;\;\;\pi_{k}=\frac{\partial 𝓛}{\partial {\dot{\xi }}_{k}}\;.$$

The value function (14) satisfies the Hamilton-Jacobi equation

(16) $$\frac{\partial v}{\partial t}\;+\;𝓗(\xi ,-\frac{\partial v}{\partial \xi })\;=\;0\;,\;\;\;\;\;\;\mathrm{with}\;\;v\left(\xi ,0\right)=0\;.$$

Along extremals, $\;\pi_{k}=-\partial v/\partial \xi_{k}$, and hence we obtain the reduced equations

(17) $$\frac{\partial 𝓛}{\partial {\dot{\xi }}_{k}}\left(\xi ,\dot{\xi }\right)=-\frac{\partial v}{\partial \xi_{k}}\left(\xi \right)\;\;\;\;\;\;\;\;\left(\;k=1,\ldots m\;\right)\;,$$

a closed system of *m* ordinary differential equations of first order in $\xi \left(t\right)\in R^{m}$. Our optimal closure approximates the relaxation of the system from an initial statistical state $\tilde{\rho }\left(\cdot ,;\xi_{0}\right)$ to statistical equilibrium (for which ξ=0) by the path of trial densities $\tilde{\rho }\left(\cdot ;\xi \left(t\right)\right)$ determined by the solution ξ(t) of (17) satisfying the initial condition $\xi \left(0\right)=\xi_{0}$.

The Liouville residual for a trial density (11), in which β(ξ) is determined by the mean energy constraint 〈H〉=E, is given by

$$R\;=\;\sum_{k=1}^{m}{\dot{\xi }}_{k}\;Q_{H}\left(\;A_{i}-\langle A_{k}\rangle \;\right)\;+\;\xi_{k}\left\{A_{k},H\right\}\;,$$

where $Q_{H}$ denotes the complementary orthogonal projection onto the energy shell, that is,

$$Q_{H}F=F-P_{H}F\;,\;\;\;\;\;\;\mathrm{with}\;\;P_{H}F=\frac{\langle F(H-E)\rangle }{\langle {\left(H-E\right)}^{2}\rangle }\left(H-E\right)\;.$$

From this formula it is evident that 〈R〉=0,〈R(H−E)〉=0, and hence that the dynamical diagnostic *R* is analogous to the score variable for a parametric statistical model [37]. In turn, its mean-square $\langle R^{2}\rangle$, which defines the lack-of-fit (13), is a dynamical analogue to the Fisher information [37]. The meaning of the conjugate variables $\pi_{k}$ is revealed by the calculation,

$$\pi_{k}=\frac{\partial 𝓛}{\partial {\dot{\xi }}_{k}}=\langle Q_{H}\left(A_{k}-\langle A_{k}\rangle \right)\;R\rangle =\langle A_{k}R\rangle \;,$$

which uses the fact that $𝓛(\xi ,\dot{\xi })$ depends, quadratically, on $\dot{\xi }$. Taking the moment with respect to $A_{k}$ of the Liouville equation yields

(18) $$\frac{d}{dt}\langle A_{k}\rangle \;=\;\langle \left\{A_{k},H\right\}\rangle \;+\langle A_{k}R\rangle .$$

Thus, $\pi_{k}$ is precisely the residual in the moment equation for $A_{k}$, due to replacing the exact density by the trial density. Accordingly, $\pi_{k}$ may be interpreted thermodynamically as the irreversible flux of the mean $\langle A_{k}\rangle$. The Hamilton-Jacobi analysis reveals that the flux vector, π, is the minus ξ-gradient of the value function v(ξ,t). Therefore *v* has the thermodynamic interpretation of a dissipation potential in the reduced equations [44].

A fuller explanation of this optimal closure is given in [22], where it is termed the “nonstationary closure”, since the value function is time-dependent. In this closure the dissipation vanishes at t=0 when the initial nonequilibrium statistical state coincides with a trial density; for large *t*, it approaches the associated “stationary closure”, for which the value function is time-independent.

Practical implementation of this model reduction procedure is inhibited by the difficulty inherent in solving the Hamilton-Jacobi Equation (16), which is not analytically tractable apart from very special (completely separable) cases. This impediment may be overcome by resorting to numerical optimization methods, such as are available in the optimal control literature; for instance, this approach is implemented in [25]. In the current paper, however, we instead restrict our attention to near-equilibrium relaxations, which allows us to linearize the optimization theory around equilibrium and thereby obtain a linear irreversible closure. Under that approximation the Hamilton-Jacobi equation becomes a matrix Riccati equation, whose solution may be identified with the matrix of transport coefficients that govern the dissipative reduced dynamics. Rather than develop this approximation in the abstract, we return in the next section to point vortex systems and elaborate the optimal closure in that setting.

## 4. Nonequilibrium Mean-Field Theory of Vortex Systems

We coarse-grain the *N*-vortex dynamics, for large *N*, in terms of finitely-many spatial moments of the vorticity defined by some chosen functions $A_{k}\left(x\right),\;k=1\;\ldots ,m$. The macrostate is then the ensemble mean of the vector observable $A^{N}\in R^{m}$ having components

(19) $$A_{k}^{N}\left(x_{1},\ldots ,x_{N}\right)=\frac{1}{N}\sum_{i=1}^{N}A_{k}\left(x_{i}\right)\;.$$

(Abusing the notation of the preceding section, henceforth $A_{k}^{N}$ denotes the observable on phase space, while $A_{k}$ denotes the spatial moment on physical space.) For instance, when we model the axisymmetrization of a distorted vortex ensemble centered at the origin (so that the first-order moments vanish and are able to be ignored), we take $A_{1}=x^{2}-y^{2},A_{2}=2xy$, along with $A_{3}=x^{2}+y^{2}$, which determines the angular impulse invariant. Heuristically, the macrostate, $a=\langle A\rangle \in R^{3}$, describes the effective “size”, “eccentricity” and “inclination” of the evolving vortex ensemble conceived as an elliptical structure.

In this section we include the angular impulse in the resolved vector, for the sake of clarity while elaborating the mean-field theory. In the later sections, we treat the angular impulse as an invariant, like the energy, and include only non-conserved resolved variables in *A*.

### 4.1. Mean-Field Ansatz

Motivated by the established fact that the equilibrium theory of vortex systems yields an asymptotically exact mean-field theory in the appropriate continuum limit, we base our nonequilibrium theory on the following trial densities on $R^{2N}$:

(20) $${\tilde{\rho }}_{N}\left(x_{1},\ldots ,x_{N};\beta ,\xi \right)=\prod_{i=1}^{N}\tilde{\zeta }\left(x_{i};\beta ,\xi \right)$$

where $\tilde{\zeta }\left(x;\beta ,\xi \right)$ is the probability density on $R^{2}$ defined by the mean-field equation

(21) $$\tilde{\zeta }=\;-\Delta \tilde{\psi }\;=\;e^{\sum_{k=1}^{m}\xi_{k}A_{k}-\beta \tilde{\psi }-\mu }\;.$$

That is, our statistical model of the *N*-vortex system assigns independent random locations, $x_{i}$, to each of the point vortices, distributed according to $\tilde{\zeta }\left(x\right)$. This spatial density is parameterized by β, conjugate to the energy invariant, *E*, and $\xi_{1},\ldots ,\xi_{m}$ conjugate to the moments, $A_{1}\left(x\right),\ldots ,A_{m}\left(x\right)$ that define the coarse-graining; μ=μ(β,ξ) normalizes $\tilde{\zeta }$. In the limit as N→∞, the law of large numbers implies that the empirical density $\zeta^{N}\left(x\right)$, defined in (4), tends to $\tilde{\zeta }\left(x\right)$ in the weak sense of measures; in turn, the smoothing properties of the Poisson equation [41] guarantee that the corresponding streamfunction $\psi^{N}\left(x\right)$, defined in (5), tends to $\tilde{\psi }\left(x\right)$, as do its first derivatives and hence the corresponding velocity field.

The trial densities (20) are constructed from solutions $\tilde{\psi }\left(x\right)$ of a nonlinear elliptic partial differential Equation (21), which depends parametrically on β and ξ. Alternatively, the marginal density, $\tilde{\zeta }\left(x\right)$, is the solution of the maximum entropy problem (10) in which the single constraint on angular impulse is replaced by the family of constraints:

$$\int_{R^{2}}A_{k}\left(x\right)\zeta \left(x\right)\;dx\;=\;a_{k}\;\;\;\;\;\;\;\;\mathrm{for}\;\;k=1,\ldots ,m.$$

The constraint values, $a_{k}$ and *E*, then parameterize the maximizers $\tilde{\zeta }\left(x\right)$ rather than the associated multipliers $-\xi_{k}$ and β. Holding the energy constant, β is slaved to ξ, and hence ξ is determined by the macrostate vector *a*. The correspondence between ξ and *a* is one-to-one in a neighborhood of equilibrium, as our subsequent analysis will show.

We submit this mean-field trial density to the Liouville equation governed by the Hamiltonian $H^{N}$ displayed in (6) (with $\gamma_{i}=1/N$), and the Poisson bracket (7). The scaled residual is

(22) $$\begin{array}{ccc} R & = & \frac{1}{N}\left(\;\partial_{t}+\left\{\cdot ,H^{N}\right\}\;\right)\log{\tilde{\rho }}_{N}\left(x_{1},\ldots ,x_{N};\beta ,\xi \right)\\ & = & \frac{1}{N}\sum_{i=1}^{N}\left(\;\partial_{t}+\left[\;\cdot ,\psi_{i}^{N}\left(x_{i}\right)\;\right]\;\right)\left(\sum_{k=1}^{m}\xi_{k}A_{k}\left(x_{i}\right)\;-\;\beta \tilde{\psi }\left(x_{i}\right)\;-\mu \left(\beta ,\xi \right)\;\right)\\ & = & \frac{1}{N}\sum_{i=1}^{N}\;\;\sum_{k=1}^{m}{\dot{\xi }}_{k}\left(A_{k}\left(x_{i}\right)-\langle A_{k}\rangle \right)+\xi_{k}\left[A_{k},\psi_{i}^{N}\right]\left(x_{i}\right)\\ & & \;\;\;\;\;\;\;\;\;\;\;\;-\dot{\beta }\left(\tilde{\psi }\left(x_{i}\right)-\langle \tilde{\psi }\rangle \right)-\beta \partial_{t}\left(\tilde{\psi }\left(x_{i}\right)-\langle \tilde{\psi }\rangle \right)-\beta \left[\psi_{i}^{N},\tilde{\psi }\right]\left(x_{i}\right)\;. \end{array}$$

This calculation makes use of the desingularized streamfunction,

$$\psi_{i}^{N}\left(x\right)=-\frac{1}{2\pi N}\sum_{j\neq i}\log\left|x-x_{j}\right|\;,$$

and the identity, $\left\{F\left(x_{i}\right),H^{N}\left(x_{1},\ldots ,x_{n}\right)\right\}=\left[F,\psi_{i}^{N}\right]\left(x_{i}\right)$. In (22), and in the analysis to follow, 〈·〉 denotes expectation with respect to the density $\tilde{\zeta }$. We notice that the mean-field ansatz produces two terms that do not appear in the residual of quasi-canonical trial densities outlined in the preceding section. The term, $\beta \partial_{t}\left(\tilde{\psi }-\langle \tilde{\psi }\rangle \right)$, accounts for the implicit dependence of the streamfunction $\tilde{\psi }$ on the parameters ξ(t),β(t). The term, $\beta [\psi_{i}^{N},\tilde{\psi }]$, vanishes in the continuum limit. Both these terms reflect the relative simplicity of the mean-field ansatz, which for finite *N* imposes the product form (20) that the quasi-canonical density achieves only in the limit as N→∞.

It is evident from (22) that, in the continuum limit as N→∞, the appropriate continuum residual on which to base a mean-field optimal closure is

(23) $$\tilde{R}\left(x;\xi ,\dot{\xi },\beta ,\dot{\beta }\right)=\sum_{k=1}^{m}{\dot{\xi }}_{k}\left(A_{k}-\langle A_{k}\rangle \right)+\xi_{k}\left[A_{k},\tilde{\psi }\right]-\dot{\beta }\left(\tilde{\psi }-2E\right)-\beta \partial_{t}\tilde{\psi },$$

noticing that $\langle \tilde{\psi }\rangle =2E$, which is constant in time. This mean-field version of the Liouville residual is derived entirely from $\tilde{\zeta }$. In fact, it may be viewed as simply the residual in the Euler equations of the ansatz (21), that is,

(24) $$\tilde{R}=\left(\partial_{t}+\left[\;\cdot ,\tilde{\psi }\;\right]\right)\;\log\tilde{\zeta }.$$

In this light, the derivation of $\tilde{R}$ from the mean-field ansatz may be viewed as a justification on information-theoretic grounds for using $𝓛=\langle {\tilde{R}}^{2}\rangle /2$ as the cost function for an optimal closure formulated in physical space in terms of quasi-equilibrium vorticity fields (21). The reduced model is therefore an optimal closure with respect to the Euler equations themselves, in which the underlying point vortex dynamics and its associated entropy functional are used to deduce the trial densities and the lack-of-fit metric appropriate to the continuum limit.

### 4.2. Near-Equilibrium Linearization

Next we linearize the optimal closure theory formulated in the preceding subsection around a given equilibrium state, $\zeta_{eq}$, with corresponding streamfunction, $\psi_{eq}$. Relaxation of an initial disturbed macrostate toward this equilibrium macrostate conserves the energy, *E*, and angular impulse, $L^{2}$, and thus the near-equilibrium linearization of the optimal closure is required to respect those invariants up to linear order in the perturbation. The mean-field macrostate is

(25) $$\tilde{\zeta }=\;-\Delta \tilde{\psi }\;=\;e^{\sum_{k=1}^{m}\xi_{k}A_{k}-\alpha {\left|x\right|}^{2}-\beta \tilde{\psi }-\mu }\;,$$

and by assumption the parameter vector, $\xi \in R^{m}$, is near the origin. (The angular impulse term is no longer included in the resolved vector, since it is an invariant.) Denoting perturbations from equilibrium by primes, so that $\alpha =\alpha_{eq}-\alpha^{\prime }$, $\beta =\beta_{eq}-\beta^{\prime }$ and $\tilde{\zeta }=\zeta_{eq}+\zeta^{\prime }$, $\tilde{\psi }=\psi_{eq}+\psi^{\prime }$, we have

$$\zeta^{\prime }\;=\;\left[\;\sum_{k=1}^{m}\xi_{k}A_{k}+\alpha^{\prime }{\left(|x\right|}^{2}-L^{2})+\beta^{\prime }\left(\psi_{eq}-2E\right)-\beta_{eq}\psi^{\prime }\;\right]\;\zeta_{eq}\;+{\;O(|\xi |}^{2}).$$

Consequently the perturbation streamfunction satisfies

(26) $$\left(-\Delta +\beta_{eq}\zeta_{eq}\right)\psi^{\prime }=\left[\sum_{k=1}^{m}\xi_{k}A_{k}+\alpha^{\prime }{\left(|x\right|}^{2}-L^{2})+\beta^{\prime }\left(\psi_{eq}-2E\right)\right]\zeta_{eq}.$$

This elliptic equation along with the boundary condition, $\lim_{|x|\to \infty }\psi^{\prime }\left(x\right)=0\;$, determines the solution $\psi^{\prime }$, which depends linearly on $\xi ,\alpha^{\prime },\beta^{\prime }$. In turn, $\alpha^{\prime },\beta^{\prime }$ are determined as linear functions of the vector ξ by solving the linearized energy and angular impulse constraints,

$$\int_{R^{2}}\psi_{eq}\;\zeta^{\prime }\;dx=0,\;\;\;\;\;\;\int_{R^{2}}{\left|x\right|}^{2}\zeta^{\prime }dx=0.$$

In summary, the linearized mean-field ansatz together with the linearized invariants yields the linear expressions

(27) $$\alpha^{\prime }=\sum_{k=1}^{m}\alpha_{k}\xi_{k}\;,\;\;\;\;\beta^{\prime }=\sum_{k=1}^{m}\beta_{k}\xi_{k}\;,\;\;\;\;\psi^{\prime }=\sum_{k=1}^{m}\psi_{k}\xi_{k}\;,$$

in which the sensitivities, denoted by $\alpha_{k}$, $\beta_{k}$, $\psi_{k}$, to the perturbation $\xi_{k}$ are calculable from the given equilibrium state $\zeta_{eq}$. We refrain from displaying explicitly the coefficient matrices involved; full details are given in [45].

The Liouville residual is, to leading order,

(28) $$R={\dot{\xi }}^{T}U+\xi^{T}V.$$

where we define the vectors U and V by

(29) $$\begin{array}{cc} U_{k} & =A_{k}+\alpha_{k}{\left(|x\right|}^{2}-L^{2})+\beta_{k}\left(\psi_{eq}-2E\right)-\beta_{eq}\psi_{k},\\ V_{k} & =\left[\;A_{k},\psi_{eq}\;\right]-\alpha_{eq}{\left[\;|x\right|}^{2},\psi_{k}\;]. \end{array}$$

Squaring and taking the expectation we obtain the quadratic lack-of-fit Lagrangian,

(30) $$2𝓛\left(\xi ,\dot{\xi }\right)={\langle R^{2}\rangle }_{eq}={\dot{\xi }}^{T}{\langle UU^{T}\rangle }_{eq}\dot{\xi }+2\xi^{T}{\langle VU^{T}\rangle }_{eq}\dot{\xi }+\xi^{T}{\langle VV^{T}\rangle }_{eq}\xi \;,$$

whose matrices

(31) $$C={\langle UU^{T}\rangle }_{eq}\;,\;\;\;\;J={\langle VU^{T}\rangle }_{eq}\;,\;\;\;\;K={\langle VV^{T}\rangle }_{eq}\;,$$

are computed from the given equilibrium state $\zeta_{eq}$.

The optimal closure thus becomes a quadratic programming problem and its Hamilton-Jacobi Equation (16) becomes a Riccati matrix equation for the m×m matrix, M(t), that defines the quadratic value function, $v\left(\xi ,t\right)=\xi^{T}M\left(t\right)\xi /2\;$. Specifically, the relations (15) become

$$𝓗(\xi ,\pi )=\frac{1}{2}\pi^{T}C^{-1}\pi -\xi^{T}JC^{-1}\pi -\frac{1}{2}\xi^{T}D\xi \;,\;\;\;\;\;\;\pi =C\dot{\xi }+J^{T}\xi \;,$$

where $D=K-JC^{-1}J^{T}$. Substituting π=−Mξ into the Hamilton-Jacobi equation then yields the following initial value problem for the matrix-valued function M(t):

(32) $$\dot{M}+MC^{-1}M+JC^{-1}M+MC^{-1}J^{T}=D,\;\;\;\;\mathrm{with}\;\;M\left(0\right)=0,$$

Known properties of Riccati equations ensure that M(t) is symmetric semi-definite, because *C* is symmetric positive definite and *D* is symmetric semi-definite [43].

The optimal parameter path ξ(t) satisfies the linear system

(33) $$C\;\frac{d\xi }{dt}\;=\;\left(\;-J^{T}-M\left(t\right)\;\right)\;\xi \;,$$

which is the linearization of (17). Since the macrostate vector *a* and the parameter vector ξ are related by

$$a\left(t\right)=\int_{R^{2}}A\left(x\right)\;\tilde{\zeta }\left(x,t\right)\;dx\;=\;C\xi \left(t\right)+{O(|\xi |}^{2})\;,$$

the reduced Equation (33) is equivalently a relaxation equation for the macrostate. This completes the optimal closure. We emphasize that this linearized, near-equilibrium relaxation equation is constructed entirely from the given equilibrium mean-field state, $\zeta_{eq}$, and is free of any adjustable constants, such as relaxation rates. Moreover, the reduced dynamics is memoryless in the macrostate a(t), although it is non-autonomous due to the time dependence of M(t).

The remainder of the paper applies this optimal closure to two simplified models arising in geophysical fluid dynamics. These models are straightforward generalizations of the two-dimensional Euler equations, and the analysis given above extends to them without any fundamental changes. We therefore concentrate on displaying and interpreting the predictions of the closure, and testing those predictions against benchmarks computed by direct numerical simulations of the many-vortex systems.

## 5. Axisymmetrization in Barotropic Quasi-Geostrophic Dynamics

The single-layer quasi-geostrophic dynamics is governed by the transport equation for the potential vorticity, denoted by q(x,t):

(34a) $$\begin{array}{cc} \partial_{t}q+\left[q,\psi \right] & =0, \end{array}$$

(34b) $$\begin{array}{cc} -\Delta \psi +R_{d}^{-2}\psi & =q. \end{array}$$

We refer the reader to the literature on geophysical fluid dynamics for the meaning of this system, and its derivation as an asymptotic model for a rotating shallow fluid layer in the limit of small Rossby number [46,47,48]. This model involves a length scale, $R_{d}$, the Rossby deformation radius, which characterizes the scale at which kinetic and potential energies balance. An ensemble composed of *N* concentrations of potential vorticity, namely,

$$q\left(x,t\right)=\sum_{i=1}^{N}\frac{1}{N}\delta \left(x-x_{i}\right)$$

interacts with a screened potential; specifically, the Green function for (34b) is the Bessel function $\left(1/2\pi \right)K_{0}\left(r/R_{d}\right)$. The Euler equation is recovered in the limit as $R_{d}\to \infty$.

The presence of a length scale in the interaction potential for the vortex system allows us to investigate how the relaxation of an ensemble of vortices depends on the ratio between $R_{d}$ and the average “radius” of the ensemble, which thanks to the angular impulse constraint is *L* for an ensemble centered at the origin. To do so we study the evolution of ensembles of vortices that are initially distorted away from a given axisymmetric equilibrium state. We measure this distortion by the second moments of the vorticity field. Besides simplicity, the justification for this choice is that a centered Gaussian distribution is completely characterized by it second moments; and equilibrium macrostates for β=0 are Gaussians. The moment of ${\left|x\right|}^{2}=x^{2}+y^{2}$ being a conserved quantity, we declare the observables

(35) $$A_{1}\left(x\right)=x^{2}-y^{2},\;A_{2}\left(x\right)=2xy\;,$$

to be the resolved variables, noticing that $A_{1}\left(x\right),A_{2}\left(x\right)$ and ${\left|x\right|}^{2}$ form an orthogonal basis for the (centered) quadratics in x with respect to any axisymmetric density, and hence any equilibrium macrostate. The ensemble mean values, $a_{1}=\langle A_{1}\rangle$, $a_{2}=\langle A_{2}\rangle$, relax to zero as the ensemble equilibrates. We normalize the spatial scale by setting $L^{2}=2$, so that a symmetric Gaussian density has unit variance in both *x* and *y*. The ensemble evolution is initialized by a centered Gaussian density with given $a_{1},a_{2}$.

In qualitative terms, an elliptically distorted initial state develops spiral arms, which are entrained and sheared by the large-scale rotation and eventually relax toward the axisymmetric equilibrium state. Our severe coarse-graining does not resolve the spiral arm structure, but instead fits trial densities to it that track its “shape”, quantified by the mean observables, $\langle A_{1}\rangle ,\langle A_{2}\rangle$ along with the angular impulse $\langle |x{|}^{2}\rangle$, and its “concentration”, controlled by the energy 〈ψ〉/2.

**Figure 2:**
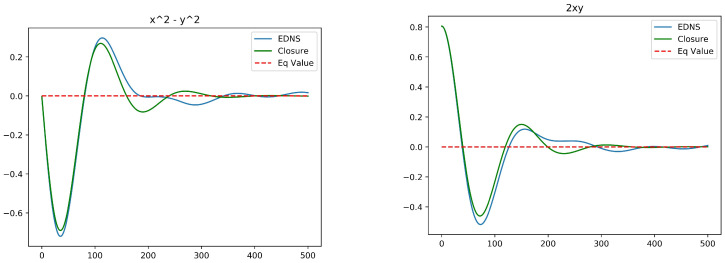
Ensemble Direct Numerical Simulation compared to the optimal closure for the Euler equations, $R_{d}=\infty$. (**left**): $a_{1}\left(t\right)=\langle x^{2}-y^{2}\rangle$; (**right**): $a_{2}\left(t\right)=\langle 2xy\rangle$. The initial values are $a_{1}\left(0\right)=0.0$ and $a_{2}\left(0\right)=0.8$.

**Figure 3:**
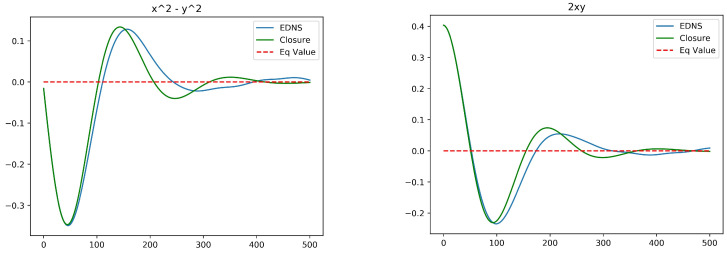
Ensemble Direct Numerical Simulation compared to optimal closure for single-layer quasi-geostrophic equations with $R_{d}=4.0$. (**left**): $a_{1}\left(t\right)=\langle x^{2}-y^{2}\rangle$; (**right**): $a_{2}\left(t\right)=\langle 2xy\rangle$. The initial values are $a_{1}\left(0\right)=0.0$ and $a_{2}\left(0\right)=0.4$.

Figure 2 plots the temporal profiles of the two mean resolved variables for Euler dynamics ($R_{d}=\infty$). The initial distortion is relatively large in this case, with $a_{1}=0,\;a_{2}=0.8$. Nonetheless, a good agreement with Ensemble Direct Numerical Simulation (EDNS) of 1000 point vortices is exhibited. In the later stages of the evolution some departures are apparent, but these can be mainly attributed to finite-*N* fluctuations. Figure 3 shows the analogous plots for $R_{d}=4$, now with $a_{1}=0,\;a_{2}=0.4$. Comparison to the full simulation of the vortex ensemble shows that the optimal closure is less accurate as $R_{d}$ is decreased. Nonetheless, it does capture the time scale of relaxation, which is longer for larger $R_{d}$. This dependence of the rate of relaxation on $R_{d}$ is attributed to the effect of screening the vortex-to-vortex interactions.

**Figure 4:**
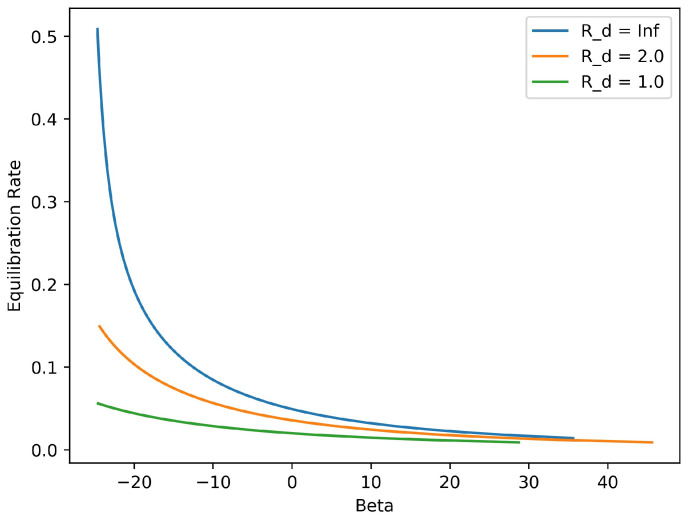
Predicted symmetrization rate versus inverse temperature β for three values of the deformation radius. As β→+∞, the equilibration rate tends to zero, indicating that symmetrization is suppressed in the deterministic limit.

**Figure 5:**
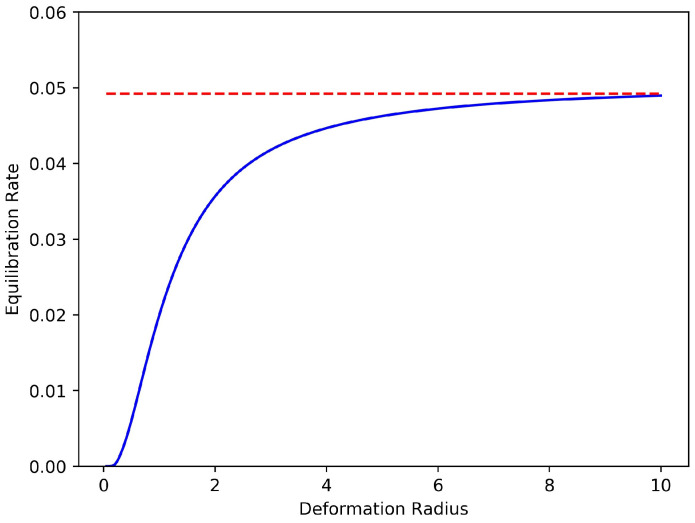
Predicted symmetrization rate versus deformation radius for β=0. The dashed red line is the equilibration value for the Euler equations, $R_{d}=\infty$.

The dependences of the rate of equilibration on the inverse temperature $\beta_{eq}$ and on the Rossby radius $R_{d}$ are displayed in Figure 4 and Figure 5, respectively. The rate plotted in both of these figures is the double eigenvalue of the 2×2 matrix M(+∞), the stationary limit of M(t) for large time *t*. That M(t) is a scalar matrix (diagonal with equal entries) is a consequence of a rotational symmetry in this reduced model: expressed in polar coordinates the two resolved variables satisfy, $A_{2}\left(r,\theta \right)=A_{1}\left(r,\theta +\pi /4\right)$. This symmetry implies that the coefficient matrices in the Riccati Equation (32) are special: *C* and *D* are scalar, and *J* is antisymmetric. (We omit the elementary calculations needed to check these properties.) It follows that the solution M(t) of (32) is necessarily a scalar matrix.

The scalar M(t) has a definite interpretation as the dissipation rate for the reduced equations. To justify this claim we consider the relative entropy $D_{KL}\left(\zeta_{eq}\;∥\;\tilde{\zeta }\right)$, which is the information distance between the nonequilibrium and equilibrium states. Under the near-equilibrium linearization, $D_{KL}\left(\zeta_{eq}\;∥\;\tilde{\zeta }\right)\approx \xi^{T}C\xi /2$. The closed reduced dynamics (33) then yields

$$\frac{d}{dt}\frac{1}{2}\xi^{T}C\xi \;=\;\xi^{T}\;\left(J-M\right)\;\xi \;=\;-\xi^{T}M\xi \;<0\;.$$

Thus the information distance decays at a rate that equals twice the value function, and hence we naturally identify M(t) as the rate of equilibration. This rate is itself time-dependent, since M(t) evolves from M(0)=0 to its stationary value M(+∞), which solves the associated algebraic Riccati equation. We therefore choose the limiting value of M(t) as t→+∞ to define the dissipation rate; this stationary value is plotted in Figure 4 and Figure 5.

Figure 4 shows that the equilibriation rate declines to zero as $\beta_{eq}\to +\infty$. This result has an interesting interpretation. In this zero-temperature limit, the equilibrium macrostates of the mean-field theory become deterministic vortex patches (flows having constant potential vorticity inside a closed boundary curve, and zero outside); this limiting property is easily demonstrated from the mean-field equation. For the Euler equations ($R_{d}=\infty$) an initially elliptical vortex patch does not axisymmetrize, being instead a rotating steady state known as a Kirchhoff ellipse (see 159 of [27]). Thus, our nonequilibrium mean-field theory is compatible with this classical deterministic result in the zero-temperature limit. The fact that the coherent structures observed in two-dimensional turbulence show a strong tendency to axisymmetrize therefore leads us to conclude that typical end states of freely decaying turbulence resemble mean-field equilibria having β<0, not large positive β.

Figure 5, in which $\beta_{eq}=0$, displays the increasing rate of equilibration with increasing $R_{d}$. This dependence is quite strong when $R_{d}$ is comparable to the size of the vortex ensemble, that is, when $R_{d}∼1$.

These results illustrate the usefulness of a severely coarse-grained model designed to omit the details of the reorganizing vorticity field, but to capture key features of its nonequilibrium behavior. In particular, the simplicity of this optimal closure allows us to define a unique rate for the axisymmetrization phenomenon, and to predict its dependence on both the temperature of the equilibrium state and the deformation radius.

Taking a broader view, the main output of the optimal closure is the matrix M(t), which controls the dissipative structure of the reduced equations. The stationary matrix M(+∞) plays the role of the transport matrix familiar from classical linear irreversible thermodynamics, in the sense that after the transcient stage it relates the thermodynamic “forces” ξ to the thermodynamic “fluxes” π [44]. From this perspective, the reduced model tested in this section may be regarded as one instantiation of a nonequilibrium thermodynamics of isolated coherent structures.

## 6. Barotropization in Two-Layer Quasi-Geostrophic Dynamics

Finally we apply our optimal closure to a simple model in geophysical fluid dynamics that includes a vertical stratification. Specifically, we consider vortex ensembles governed by the two-layer quasi-geostrophic equations [47,48]. For simplicity, we assume that the two layers have equal depth, and are bounded by a flat bottom and a flat top; the fluid in the lower layer is denser than the upper layer. These shallow layers are separated by a free surface, and their interaction is controlled by a single parameter, $R_{d}$, the internal Rossby deformation radius. In the appropriate quasi-geostrophic limit, the continuum equations for this system reduce to two coupled transport equations for the potential vorticity q(x,t) in each layer; the upper layer is indexed by 1, the lower layer by 2:

(36a) $$\begin{array}{c} \partial_{t}q_{1}+\left[q_{1},\psi_{1}\right]=0,\;\;\;\;\;\;q_{1}=-\Delta \psi_{1}+R_{d}^{-2}\left(\psi_{1}-\psi_{2}\right)\;, \end{array}$$

(36b) $$\begin{array}{c} \partial_{t}q_{2}+\left[q_{2},\psi_{2}\right]=0,\;\;\;\;\;\;q_{2}=-\Delta \psi_{2}-R_{d}^{-2}\left(\psi_{1}-\psi_{2}\right)\;. \end{array}$$

Direct numerical simulations of rotating stratified fluids [11] reveal a tendency for coherent vorticity structures to approach purely barotropic end states, for which $q_{1}\approx q_{2}$. This “barotropization” reflects a preference for minimal potential energy states. As such it is similar to baroclinic instability, in that it is a mechanism to convert available potential energy into kinetic energy [47,48].

In light of the physical significance of this tendency toward barotropic states, we now employ our optimal closure theory to predict the rate at which initially baroclinic perturbations return to stable barotropic states. Our analysis may be viewed as complementary to baroclinic instability theory, in that we predict the rate of conversion of available potential energy into kinetic energy during the end stages of a baroclinic evolution whereas linear stability analysis treats its initial stages.

We examine only the simplest formulation of this general question. Namely, we consider two like-signed vortical structures in the two layers, and demand that they have equal circulation, $\Gamma_{1}=\Gamma_{2}=1$. A barotropic state is one in which the two vortical structures are vertically aligned. We therefore create a baroclinic initial state by horizontally displacing the centers of potential vorticity in the two layers. The mutual advection of these tilted structures as they realign into a barotropic state may be viewed as a three-dimensional analogue to the axisymmetrization of a single-layer vortical structure from an elliptically distorted state, examined in the preceding section.

We take the resolved vector for this problem to be the separation of the centers of the vortex ensembles in the two layers. In the notation of the previous sections, the ensemble-averaged macrostate *a* has the components

$$a_{1}\left(t\right)=\int_{R^{2}}x\;\left(\;q_{1}\left(x,t\right)-q_{2}\left(x,t\right)\;\right)\;dx\;,\;\;\;\;\;\;a_{2}\left(t\right)=\int_{R^{2}}y\;\left(\;q_{1}\left(x,t\right)-q_{2}\left(x,t\right)\;\right)\;dx\;.$$

Thus, the macrostate consists of the first-order spatial moments of the baroclinic part of *q*, while the barotropic first-order moments, $\int x(q_{1}+q_{2})\;dx=0$, are conserved. The conserved total energy and angular impulse are, respectively,

$$\frac{1}{2}\int_{R^{2}}\left(\;\psi_{1}q_{1}\;+\;\psi_{2}q_{2}\;\right)\;dx\;=\;E\;,\;\;\;\;\;\;\int_{R^{2}}{\left|x\right|}^{2}\;\left(\;q_{1}+q_{2}\;\right)\;dx\;=\;L^{2}\;.$$

Unlike our single-layer reduced models, the second-order moments apart from the $L^{2}$ are not included in the resolved macrostate. Accordingly, our model is the severest reduction of the two-layer dynamics: a mean-field theory that includes only first-order spatial moments of potential vorticity along with exact invariants.

The lack-of-fit Lagrangian is derived from a mean-field ansatz that treats the ensembles of vortices in each layer as independent. We omit the straightforward but lengthy calculations needed to formulate this optimal closure for the two-layer dynamics, preferring instead to present some predictions and tests of the ensuing reduced model; details are available in [45].

**Figure 6:**
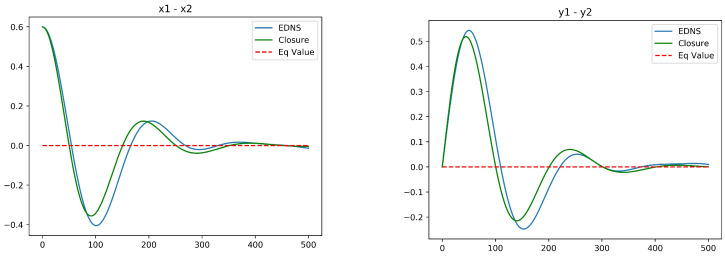
EDNS compared to optimal closure for two-layer quasi-geostrophic dynamics for $R_{d}=1.0$. (**left**): $a_{1}\left(t\right)=\langle x_{1}-x_{2}\rangle$; (**right**): $a_{2}\left(t\right)=\langle y_{1}-y_{2}\rangle$. The initial conditions are $a_{1}=0.6$ and $a_{2}=0.0$.

Figure 6 compares the optimal closure for initial conditions $a_{1}=0.6,a_{2}=0.0$ with $R_{d}=1.0$ against EDNS of a potential vortex system with 1000 vortices in each layer. The initial state consists of Gaussian densities in each layer with $L^{4}=4$, chosen so that a Gaussian barotropic state has *x* and *y* variances in each layer normalized to unity. As the horizontal separation between the center of potential vorticity in each layer is increased in the initial state, the *x* and *y* variances are correspondingly decreased to maintain the total angular impulse. Since the size of the vortex ensembles is near to $R_{d}=1$, there is strong interaction between the layers and the evolving structure reorganizes into a barotropic equilibrium state relatively quickly. Even a moderate increase to $R_{d}=2$, however, results in a slower equilibration and poorer agreement between the EDNS and the optimal closure. The source of this disagreement may be the crudeness of the closure based only on spatial first moments, or the linearization of the closure around equilibrium. It may also be related to memory effects, which are absent from the mean-field closure used throughout this paper.

**Figure 7:**
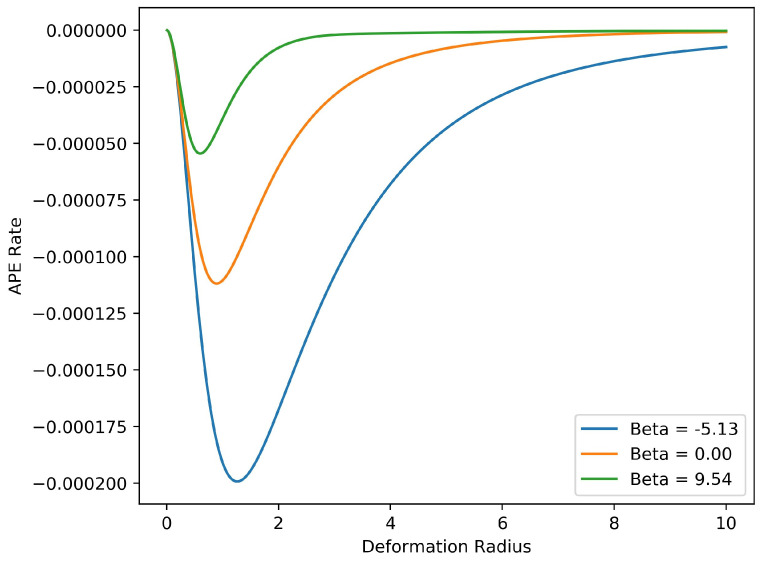
Predicted conversion rate of available potential energy as a function of deformation radius $R_{d}$ for several values of inverse temperature β. The peak transfer rates occur when the deformation radius is near to the size of the vortex ensemble.

Figure 7 displays the dependence of the rate of energy transfer from potential to kinetic energy on the inverse temperature $\beta_{eq}$ of the barotropic equilibrium state, and on the internal deformation radius $R_{d}$. The available potential energy is expressible as a quadratic form in $\xi \in R^{2}$, namely,

$$APE=\frac{1}{2R_{d}^{2}}\int_{R^{2}}{\left(\psi_{1}^{\prime }-\psi_{2}^{\prime }\right)}^{2}\;dx\;=\;\frac{1}{2}\xi^{T}P\xi \;,$$

where *P* is the matrix with elements,

$$P_{k\ell }=\frac{1}{R_{d}^{2}}\int_{R^{2}}\left(\psi_{1k}^{\prime }-\psi_{2k}^{\prime }\right)\;\left(\psi_{1\ell }^{\prime }-\psi_{2\ell }^{\prime }\right)\;dx\;;$$

we recall the notation introduced in (27). Under the closed reduced dynamics, therefore, the rate of conversion of energy is given by

$$\frac{d}{dt}APE\;=\;\xi^{T}P\frac{d\xi }{dt}=\xi^{T}PC^{-1}\left(J-M\right)\xi \;.$$

As in the discussion of the single-layer equilibration rate, the 2×2 matrices involved in this equation are special, due to the spatial symmetries of our reduced model; namely, *C*, *P* and *M* are scalar matrices, while *J* is antisymmetric. The factor $-PC^{-1}M$ therefore controls the rate of conversion of APE, and accordingly this scalar factor is displayed in Figure 7.

The APE conversion factor is fastest for β<0; these barotropic end states are concentrated coherent structures, and for increasing negative β their potential vorticity increasingly concentrates at the origin. For β>0, the APE conversion factor is noticeably smaller, becoming vanishingly small for large positive β. For each fixed β, the fastest conversion occurs when $R_{d}$ nearly equals the horizontal extent of the vortex ensemble, which is normalized to near unity in our tests. This behavior of the reduced model is reminiscent of linear stability analysis, which identifies the most active modes as those with length scales close to the deformation scale [47,48]. In this regime our optimal closure successfully follows the relative displacement of potential vorticity in the two layers and captures the associated energy transfer. Outside of this regime the barotropization process is slower and the reduced model is less accurate. Presumably this model is too severely coarse-grained to represent the saturation of a baroclinic perturbation accurately over a wide range of deformation scales. Nonetheless, it is the simplicity of the model that allows us define a characteristic rate of energy conversion and thus to identify the conditions under which barotropization proceeds most efficiently.

## 7. Conclusions

The optimal closure method used in this paper to coarse-grain point vortex dynamics is a systematic approach to model reduction. From a deterministic microscopic dynamics and a chosen macroscopic description, it invokes an information-theoretic metric to characterize that macroscopic evolution which best fits the underlying microdynamics. The metric quantifies the rate of information loss due to statistical reduction onto the macroscopic observables. The closed reduced equations derived from this intrinsic criterion rely on no intermediate stochastic modeling or adjustable closure parameters. Practical implementation of the optimal closure, however, may be computationally burdensome, because it requires dynamical optimization over paths of macrostates.

In the present application we have restricted our attention to relaxation from near-equilibrium perturbations, which allows us to linearize the optimal closure around equilibrium states, thereby making it computationally efficient. Motivated by the self-organization of coherent vortex structures observed in two-dimensional turbulence, we have derived closed reduced equations for the late stage of this organization. Our closure retains only a few spatial moments of the vorticity, making it a severe coarse-graining of the vortical structure. Comparisons against fully-resolved computations of systems containing 1000 point vortices have shown that the optimal closure approximates the temporal behavior of the few observables retained in the coarse-grained description, and that it furnishes a good estimate of the rate of relaxation toward equilibrium.

The dependence of the relaxation rate on physical parameters is more striking when the fluid dynamics is extended from the two-dimensional Euler equation to the quasi-geostrophic equations for either the single-layer or two-layer shallow water models. In these geophysical models, the Rossby radius of deformation, $R_{d}$ (external or internal depending on the model), is the key length scale. We have therefore tested how the reduced models of these quasi-geostrophic vortex systems compare against full simulations over a range of deformation scales. The axisymmetrization of single-layer structures has been shown to proceed at rates that decrease with decreasing $R_{d}$. The reduced model based on second-order spatial moments approximates the relaxation toward axisymmetry most accurately for large $R_{d}$, showing less accuracy as $R_{d}$ decreases and the vortex-vortex interactions weaken. In the two-layer situation, the reduced model, which resolves only the displacement of the centers of vorticity between the two layers, predicts the decay of the baroclinic part of the mean flow during relaxation toward a stable barotropic mean flow. We have found that this reduced model performs best when the spatial scale of the vortical structure is near to $R_{d}$, which is the regime in which the transfer of potential energy into kinetic energy is the fastest. Even though this result pertains to the late stage of relaxation toward a stable structure, it harmonizes with the intuition drawn from classical linear analysis of baroclinic modes in the early stage of an instability, in which the fastest growing modes are those whose scale is comparable to $R_{d}$.

The reduced models implemented in this paper have been designed to represent the gross spatial features of isolated vortical structures, and have therefore been based on a severe coarse-graining that uses only low-order spatial moments of the vorticity field. Nonetheless, we have presented the nonequilibrium mean-field theory of vortex systems for an arbitrary spatial coarse-graining. This general derivation naturally suggests that the theory could be applied to more refined reduced models having more resolved variables, $A_{1}\left(x\right),\ldots ,A_{m}\left(x\right)$. For instance, one could consider vortex systems whose evolving macroscopic states consist of more than one “patch” of vorticity, and design a set of basis functions, $A_{k}\left(x\right)$, that coarsely represent the location of those patches. In particular, a nonequilibrium model of the merger (or coalescence) of two such patches, which is observed as a typical phenomenon in two-dimensional turbulence [12,49], could be attempted. Implementation of the optimal closure under these conditions, however, would be beyond the reach of linearization around equilibrium and would therefore require numerical optimization procedures, such as those used in optimal control. Moreover, a memoryless closure in terms of mean-field trial densities might be too crude to capture such far-from-equilibrium behavior. The development of optimal closures having wider scope than those developed in this paper, therefore, presents challenges for future research.
